# Supplementary material for: Developing a data repository to support interdisciplinary research into childhood stunting: a UKRI GCRF Action Against Stunting Hub protocol paper
Source: BMJ Paediatr Open. 2024 Jun 5;8(Suppl 1):e002443. doi: 10.1136/bmjpo-2023-002443 (PMC11163590; doi:10.1136/bmjpo-2023-002443)
Supplement: Author's manuscript [file bmjpo-2023-002443.draft_revisions.pdf]

BMJ Paediatrics Open

BMJ Paediatrics Open is committed to open peer review. As part of this commitment we make the peer review history of every article we publish publicly available.

When an article is published we post the peer reviewers' comments and the authors' responses online. We also post the versions of the paper that were used during peer review. These are the versions that the peer review comments apply to.

The versions of the paper that follow are the versions that were submitted during the peer review process. They are not the versions of record or the final published versions. They should not be cited or distributed as the published version of this manuscript.

BMJ Paediatrics Open is an open access journal and the full, final, typeset and author-corrected version of record of the manuscript is available on our site with no access controls, subscription charges or pay-per-view fees (http://bmjpaedsopen.bmj.com).

If you have any questions on BMJ Paediatrics Open's open peer review process please email info.bmjpo@bmj.com

BMJ Paediatrics Open

Developing a data repository to support interdisciplinary research into childhood stunting: a UKRI GCRF Action Against Stunting Hub protocol paper

Journal:	*BMJ Paediatrics Open*
Manuscript ID	bmjpo-2023-002443
Article Type:	Protocol
Date Submitted by the Author:	11-Dec-2023
Complete List of Authors:	Conway-Moore, Kaitlin; London School of Hygiene & Tropical Medicine, Health Services Research and Policy<br>Tata, Darius; London International Development Centre (LIDC), London, UK<br>Wood, Peter; Birkbeck University of London<br>Katerinchuk, Val; Birkbeck University of London<br>Yadav, Dinesh; London School of Hygiene & Tropical Medicine, Department of Population Health<br>Augustine, Little Flower; ICMR-National Institute of Nutrition<br>Munikumar, Manne; ICMR-National Institute of Nutrition,<br>Diop, Assana; Service de Parasitologie-Mycologie- Pédiatrie, Faculté de médecine, Université Cheikh Anta Diop (UCAD), Dakar, Senegal<br>Tairou, Fassiatou; Service de Parasitologie-Mycologie- Pédiatrie, Faculté de médecine, Université Cheikh Anta Diop (UCAD), Dakar, Senegal<br>Jobarteh, Modou; London School of Hygiene & Tropical Medicine, Epidemiology & Population Health<br>Kulkarni, Bharati; National Institute of Nutrition<br>Faye, Babacar; Université Cheikh Anta Diop (UCAD)<br>Haggarty, Paul; University of Aberdeen<br>Heffernan, Claire; London School of Hygiene and Tropical Medicine, Department of Population Health
Keywords:	Data Collection, Growth, Physiology

SCHOLARONE™
Manuscripts

I, the Submitting Author has the right to grant and does grant on behalf of all authors of the Work (as defined in the below author licence), an exclusive licence and/or a non-exclusive licence for contributions from authors who are: i) UK Crown employees; ii) where BMJ has agreed a CC-BY licence shall apply, and/or iii) in accordance with the terms applicable for US Federal Government officers or employees acting as part of their official duties; on a worldwide, perpetual, irrevocable, royalty-free basis to BMJ Publishing Group Ltd ("BMJ") its licensees and where the relevant Journal is co-owned by BMJ to the co-owners of the Journal, to publish the Work in this journal and any other BMJ products and to exploit all rights, as set out in our licence.

The Submitting Author accepts and understands that any supply made under these terms is made by BMJ to the Submitting Author unless you are acting as an employee on behalf of your employer or a postgraduate student of an affiliated institution which is paying any applicable article publishing charge ("APC") for Open Access articles. Where the Submitting Author wishes to make the Work available on an Open Access basis (and intends to pay the relevant APC), the terms of reuse of such Open Access shall be governed by a Creative Commons licence – details of these licences and which Creative Commons licence will apply to this Work are set out in our licence referred to above.

Other than as permitted in any relevant BMJ Author's Self Archiving Policies, I confirm this Work has not been accepted for publication elsewhere, is not being considered for publication elsewhere and does not duplicate material already published. I confirm all authors consent to publication of this Work and authorise the granting of this licence.

Confidential: For Review Only

Developing a data repository to support interdisciplinary research into childhood stunting: a UKRI GCRF Action Against Stunting Hub protocol paper

Authors

Kaitlin Conway-Moore[1], Darius Tetsa Tata[2], Peter Wood[3], Val Katerinchuk[3], Dinesh Yadav D M[2], Little Flower Augustine[4], Manne Munikumar[4], Assana Diop[5], Fassiatou Tairou[5], Modou Lamin Jobarteh[2], Bharati Kulkarni[4], Babacar Faye[5], Paul Haggarty[6] and Claire Heffernan[2]

Affiliations

[1]Department of Health Services Research and Policy, London School of Hygiene and Tropical Medicine, London, United Kingdom

[2]Department of Population Health, London School of Hygiene and Tropical Medicine, London, United Kingdom

[3]Department of Computer Science and Information Systems, Birkbeck University of London, London, United Kingdom

[4] ICMR-National Institute of Nutrition, Jamai-Osmania PO, Hyderabad, India

[5]Faculté de Médecine, Pharmacie et Odontologie Université Cheikh Anta Diop de Dakar (UCAD), Dakar, Senegal

[6]Rowett Institute of Nutrition and Health, University of Aberdeen, Aberdeen, United Kingdom

Corresponding Author: Claire Heffernan, London International Development Centre, 20 Bloomsbury Square, London, UK WC1A 2NS; email: Claire.Heffernan@lidc.ac.uk

Confidential: For Review Only

Abstract

Introduction

As a topic of inquiry in its own right, data management for interdisciplinary research projects is in its infancy. Key issues include the inability of researchers to effectively query diverse data outputs and to identify potentially important synergies between discipline-specific data. Equally problematic, few semantic ontologies exist to better support data organisation and discovery. Finally, while interdisciplinary research is widely regarded as beneficial to unpacking complex problems, non-researchers such as policymakers and planners often struggle to use and interrogate the related datasets. To address these issues, the following article details the design and development of the UKRI GCRF Action Against Stunting Hub (AASH)'s All-Hub Data Repository (AHDR).

Methods and analysis

The AHDR is a single application, single authentication web-based platform comprised of a data warehouse to store data from across the AASH's three study countries and to support data querying. Four novel components of the AHDR are described in the following article: 1) a unique data discovery tool 2) a metadata catalogue that provides researchers with an interface to explore the AASH's data outputs and engage with a new semantic ontology related to child stunting; 3) an interdisciplinary aid to support a directed approach to identifying synergies and interactions between AASH data and 4) a decision support tool that will support non-researchers in engaging with the wider evidence-based outputs of the AASH.

Ethics and dissemination

Ethical approval for this study was granted by institutional ethics committees in the United Kingdom, India, Indonesia, and Senegal. Results will be disseminated via publications in peer-reviewed journals; presentations at international conferences and community-level public engagement events; key stakeholder meetings; and in public repositories with appropriate Creative Commons licences allowing for the widest possible use.

What is known about the subject

- Data management for large-scale interdisciplinary research projects can pose significant challenge to researchers.

What this study hopes to add

- Insight into designing a unique data management system, with recommendations for developing an integrated structure capable of supporting interdisciplinary analysis, storage, querying and decision support.

How this study might affect research, practice or policy

- By identifying a range of data management innovations through our own large-scale interdisciplinary research project, this work has the potential to inform the field.

Confidential: For Review Only

Introduction

Interdisciplinary research centres on the creation of new knowledge from the explicit combination of different disciplines, sub-disciplines or topics, with interdisciplinary research and the underpinning collaboration recognised to be better at unpacking complex problems and supporting innovative solutions. Despite growing attention in recent years to the merits of interdisciplinary research, however, tools for effective data management remain limited.(1,2) As a result, interdisciplinary data analysis is often done in an ad hoc manner, with researchers often left to their own devices. (3,4) Relatedly, there is little guidance in the literature on how to manage data from multiple disciplines to identify the most promising synergies and inter-relationships between such data. To address these gaps, researchers are moving to develop semantic ontologies or agreed vocabularies to search and organise data around particular topics.

While the development of semantic ontologies clearly enhances the ability of researchers to explore and analyse data across multiple disciplines within a particular field, the needs of other stakeholders of these research outputs are less well addressed. In particular, the ability of policymakers and planners to mine interdisciplinary datasets is limited. For example, there are currently a handful of widely-used global datasets related to child nutrition (e.g. WHO Global Database on Child Growth and Malnutrition, the World Bank Health Nutrition and Population Statistics, the UNICEF-WHO-World Bank Joint Child Malnutrition Estimates or indeed, the University of Oxford's Young Lives dataset), yet these tools do not explicitly focus on cross-disciplinary synergies within their data outputs.

Seeking to address these issues and inform the emerging field of interdisciplinary data management, the following article details the protocol for developing the UKRI GCRF Action Against Stunting Hub (AASH)'s All-Hub Data Repository (AHDR). The Hub's 18 institutional collaborators will collect and analyse approximately 800,000 data points from study participants in India, Indonesia and Senegal. As such, we are collecting a diverse array of data from surveys, direct observations and anthropometric measurements to focus group discussions and in-depth qualitative interviews to biological and environmental samples.

The AHDR is underpinned by three key questions:

1. What is the best platform for the storage and querying of a diverse three-country comparative dataset related to child stunting?
2. What does a new semantic ontology for child nutritional research look like?
3. Can such a data management tool also be used to create a decision-support tool relevant for diverse audiences of policymakers and planners working on child stunting globally?

A key aim of the AHDR will be to make anonymized AASH data public facing as part of the UKRI's open research data strategy, which seeks to make research processes and findings as transparent, understandable, and reproducible as possible. While access to AASH data will be restricted to project staff during the project and initial data analysis period (18-months from the project end-date), at the end of this period, all restrictions on data access will be removed and data licences updated to enable free download. In line with UKRI guidelines, this data will be

Confidential: For Review Only

accessible for 10 years after the project ends, and held on a London School of Hygiene &Tropical Medicine server for this period.

Methods and analysis

All-Hub Data Repository Design

The Data Warehouse, the Data Discovery Tool and the Interdisciplinary Aid

The AHDR data warehouse will integrate and store AASH data from the various field-level data collection tools, including CommCare, KoboCollect, REDCap and laboratory analyses using an automated Integration Platform as a Service (iPaaS) middleware system, powered by OpenFn.(5–8) The iPaaS middleware will either link to these data collection platforms directly via API connections, or to Secure File Transfer Protocol (SFTP) servers that will contain ready-to-export data in CSV or XLS formats. To ensure that there is alignment in the types of data collected across our three study countries, all metadata from our data collection applications have undergone an extensive process of labelling, mapping and review.

To prepare data stored in the data warehouse for analysis by end users, measures will be put in place for automated data cleansing both at the point of collection and during the data integration process. Specifically, data validation rules and skip logic will be implemented in different Clinical Data Management Applications (CDMAs) to reduce the risk of erroneous and inconsistent data. In addition to this, external scripts will be written in R, STATA and Python to enforce data quality checks in the event they cannot be implemented into the CDMAs, and for the purposes of anonymising/pseudo-anonymising data prior to transfer into the data warehouse. Data integration programs will also be designed to map data from across the AASH by harmonising common variable names and labels; standardising values; calculating derived variables; and recoding data as required. Data collection and transformation will be done in accordance with AASH study protocols, standard operating procedures (SOPs), General Data Protection Regulation (GDPR) guidelines and both national and local regulations for data protection in Senegal, India and Indonesia.

The data warehouse has been built using PostgreSQL, an open-source database management system that specializes in securely storing complex datasets.(9) Genomic data collected as part of our study will be held on the European Geno-phenome Archive (EGA) and connected to the AHDR using APIs. Each of our three project countries will also be equipped with high-performance computing (HPC) servers that allow for the maintenance, computing and analysis of these large files. Researchers seeking access to genomic data will be able to do so via the AHDR, following completion of consent and authorisation forms, as required. The AHDR's data warehouse will also store a limited set of derived and summary variables related to genomic data.

FIGURE 1 HERE

Figure 1: Data discovery tool for the data warehouse

Confidential: For Review Only

A Data Discovery Tool, seen in **Figure 1**, is being implemented to enable researchers and other end users to browse and download data from the data warehouse. This functionality will enable both viewing and downloading in raw CSV file format and a CSV download displaying metadata labels in place of encoded column names and multi-choice values. To facilitate the cross-disciplinary analysis, it will be possible to navigate through individual columns of the various data tables and combine them into a single CSV output, joined on a unique subject identification number, or alternative identifiers when a subject identification number is not present.

The Interdisciplinary Aid (IA) will be created by linking the various sources of data collected across our project sites to both topic-based and conceptual entities through a Neo4j graph database system (Version 4). All data variables will be tagged and allocated to a position within a defined hierarchy using specific domain ontologies. The IA will enable the establishment of direct connections between variables based on their syntactic matching with data tags and/or within the hierarchy. The tool will also enable the establishment of indirect connections between variables and datasets based on their semantic proximity (i.e., ontological connection) with the aid of semantic matching algorithms. Researchers will then have a visual interface that they can browse to narrow in on the types of data they find most useful to their work, while also finding conceptual/logical connections to data outside their primary discipline. Finally, research collaboration will also be supported via the platform's use of CKAN (Version 2.9), an open-source data portal. With CKAN, researchers will be able to upload analysed and partially analysed datasets for use by other researchers both within and outside of their discipline. In doing so, the building blocks of interdisciplinary analysis will be made available to all end users.

Meta-Data Catalogue/Semantic Ontology

The AHDR metadata catalogue will provide an overview of all the AASH data available for download and analysis. Underpinning the metadata catalogue will be a new semantic ontology for child stunting. To create the semantic ontology, first, terms from existing ontologies from across the thematic disciplines involved in the AASH (e.g., MeSH, FoodOn, ECCD Ontology) will be mined.(10,11) Second, expert consultations will take place to check, define and agree terms. The terms will then be used to organize the different classes of data collected by the AASH into a hierarchy based on their various categories, the types of relationships between them, and their potential 'nodes' or areas where disciplines overlap.(12,13)

The Decision Support Tool

The AHDR aims to provide a decision support tool to enable policymakers, practitioners and field staff to interrogate AASH data. The tool will predict a child's risk for being on the pathway to stunting. The app will prompt users for a number of input variables which will be sent back to the Python model to predict a traffic light colour. Additionally bulk-processing functionality will be available via uploading a file of input variables. Users can then download their file, modified with a predicted colour for each row of input variables in the input file. Various visualisations, including geographic visualisation, based on the bulk data, will also be provided.

Figure 2 provides an overview of the interconnected components of the AHDR as outlined above, including the ways in which it integrates inputs held in our project management and

Confidential: For Review Only

communication application (i.e., Glasscubes), with our field-data collection software (i.e., CommCare, Kobo Connect, and REDCAP), and genomic data stored on high performance computers. As seen below, all data from these sources will be integrated via our iPaaS middleware and sent to our data warehouse. The data warehouse will then feed all structured data into the web-based data portal, where researchers and other key stakeholders can interact with and export AASH data chosen from both thematic and interdisciplinary combinations. Interested parties will ultimately also be able to draw information from the data warehouse using the interdisciplinary aid and decision support tool, and our project management staff will have secure access to information related to our project's progress via the MEL Tableau dashboards.

FIGURE 2 HERE

Figure 2: Visualisation of the All-Hub Data Repository (AHDR)

Patient and public involvement

Participant and public involvement towards the eradication of child stunting is at the core of our project. We are working directly with the local communities in our study countries in India, Indonesia and Senegal, respectively, to understand how stunting and the work of the AASH has impacted their daily lives. Along with this, with a strong focus on user experience and interaction at the core of the AHDR's design, we aim for our work to bring researchers from different disciplines together to share knowledge and expertise, including allowing researchers to upload their analyzed datasets for use by others. The AHDR also aims to provide a resource for policymakers and practitioners to hone interventions that will have the highest possible benefit to the general public.

Ethics and dissemination

Ethical approval for the Action Against Stunting Hub, inclusive of the All-Hub Data Repository, was granted by the Ethics Committee of the London School of Hygiene and Tropical Medicine (17915/RR/17513); the Social Science Research Ethical Review Board at the Royal Veterinary College (URN SR2020-0197); and the International Livestock Research Institute Institutional Research Ethics Committee (ILRI-IREC2020-33). Research site-specific approvals were granted by the National Institute of Nutrition (ICMR), Ministry of Health and Family Welfare, Government of India (CR/04/I/2021); Health Research Ethics Committee, University of Indonesia and Cipto Mangunkusumo Hospital (KET-887/UN2.F1/ETIK/PPM.00.02/2019); and the Comité National d'Ethique pour la Recherche en Santé, Senegal (Protocole SEN19/78).

Dissemination will include publications in international peer-reviewed journals; community-level public engagement events, national and international conferences and key stakeholder meetings; and the use of the AHDR by our vast network of AASH researchers, and ultimately, the wider research and policymaking community. The AASH will seek to make data as open as possible as is consistent with UKRI priorities and FAIR (Findable, Accessible, Interoperable, Reusable) data principles. Upon publication, study data will be posted in public repositories (preferably domain-specific), or as required by the relevant publication body. Published data will

Confidential: For Review Only

be made available with a preference for minimal licence restrictions (typically CC BY, CC BY-NC, or CC BY-ND) as per UKRI guidance, following assessment for exploitation potential.

Authors' contributions

KCM and CH drafted the manuscript; KCM, CH, DTT, PW and VK provided critical revision to the manuscript; CH, PW, VK and DTT designed the tool, KCM, DTT, PW, VK, DYDM, LFA, MM, AD, and FT contributed to the design. All authors read and approved the final manuscript. We acknowledge and thank Taylor Downs, Aleksa Krolls, Joseph Sam and Aissatou Diallo at OpenFn who the Action Against Stunting Hub contracted to build the AHDR.

Funding statement: This work is supported by UKRI-GCRF Action Against Stunting Hub, grant number MR/S01313X/1.

Competing interests statement: The authors declare no competing interests.

References

1. Gohar F, Maschmeyer P, Mfarrej B, Lemaire M, Wedderburn LR, Roncarolo MG, et al. Driving Medical Innovation Through Interdisciplinarity: Unique Opportunities and Challenges. Front Med (Lausanne). 2019;6(35):1–6.
2. Rey J, Koplan P, Bond C, Merson MH, Reddy S, Rodriguez MH, et al. Towards a common definition of global health. The Lancet. 2009;373:1993–5.
3. Tobi H, Kampen JK. Research design: the methodology for interdisciplinary research framework. Qual Quant. 2018 May 1;52(3):1209–25.
4. Heffernan C. Building synergies in development research: Is it time for the mosaic approach? Dev Pract. 2008 Nov;18(6):686–700.
5. Dimagi. CommCare [Internet]. 2022 [cited 2022 Aug 1]. Available from: https://www.dimagi.com/commcare/
6. Kobo Inc. About KoBo [Internet]. 2022 [cited 2022 Sep 9]. Available from: https://www.kobotoolbox.org/kobo/
7. Vanderbilt. About REDCap [Internet]. 2022 [cited 2022 Sep 9]. Available from: https://projectredcap.org/about/
8. OpenFn. Automation & Data Integration [Internet]. 2022 [cited 2022 Apr 8]. Available from: https://www.openfn.org/
9. The PostgreSQL Global Development Group. What is PostgreSQL? [Internet]. 2022 [cited 2022 Sep 9]. Available from: https://www.postgresql.org/about/
10. Maedche A, Staab S. Ontology Learning for the Semantic Web. IEEE Intell Syst. 2001;16(2):72–9.
11. Noy NF. Semantic integration: A survey of ontology-based approaches. SIGMOD Rec. 2004;33(4):65–70.
12. Ontotext. What is a Knowledge Graph? [Internet]. 2022 [cited 2022 Nov 2]. Available from: https://www.ontotext.com/knowledgehub/fundamentals/what-is-a-knowledge-graph/

Confidential: For Review Only

13. Jacob EK. Ontologies and the Semantic Web. Bulletin of the American Society for Information Science and Technology. 2003 Apr 1;29(4):19–22.

Confidential: For Review Only

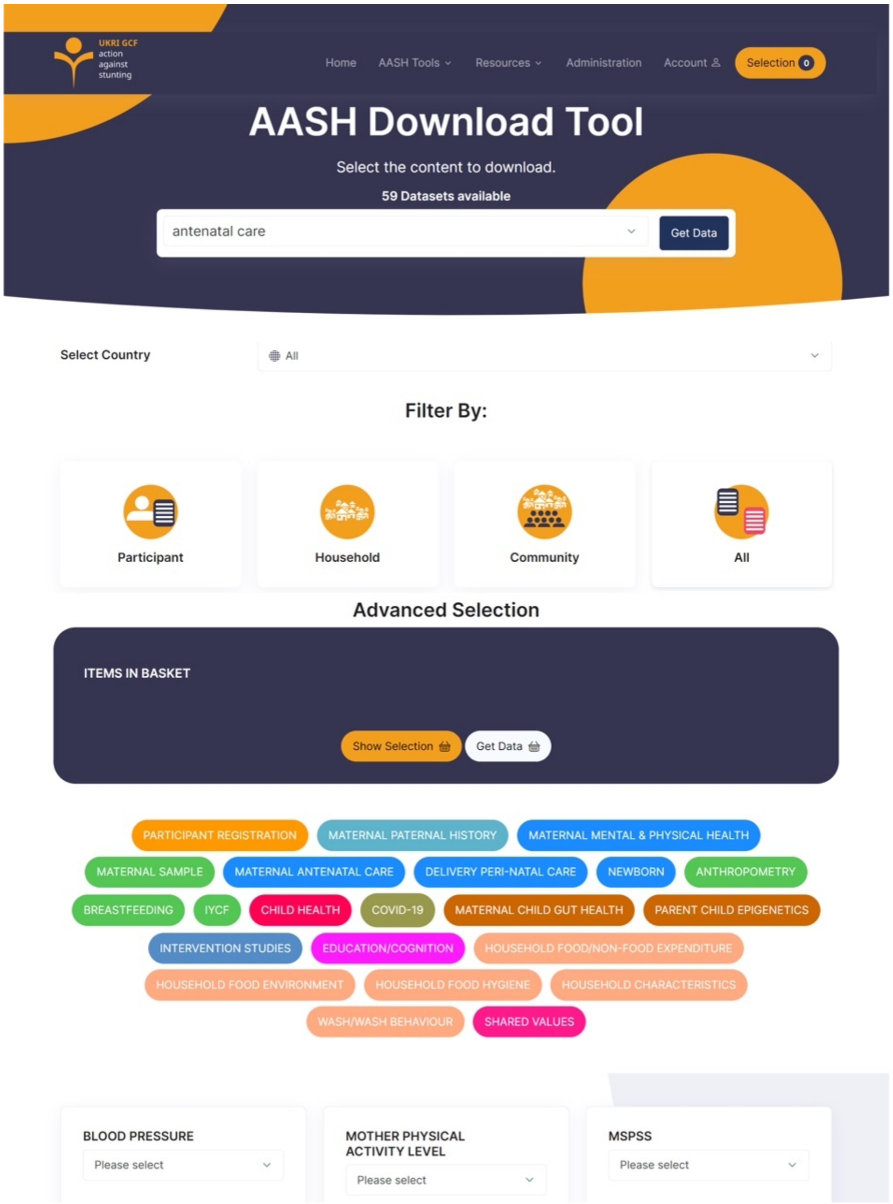

Figure 1: Data discovery tool for the data warehouse

110x148mm (220 x 220 DPI)

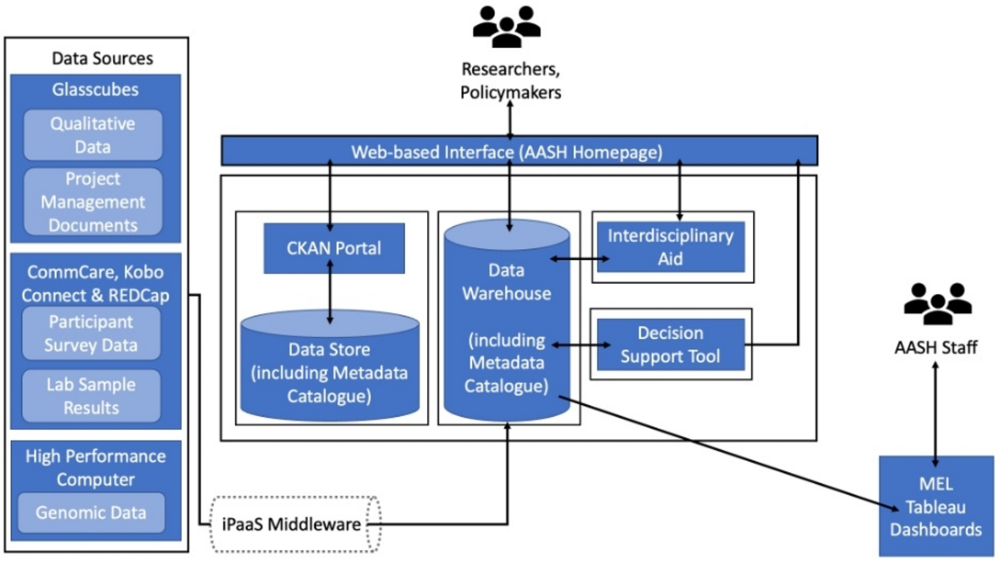

Figure 2: Visualisation of the All-Hub Data Repository (AHDR)

165x92mm (144 x 144 DPI)

Response to Editor's Comments

1. *The manuscript is includes some terminology that will be confusing to readers. For example, it is not clear what is meant by 'interdisciplinary data' – readers will know what an interdisciplinary study or method might be, but this term needs explaining or (more likely) changing since the data themselves are neither disciplinary nor interdisciplinary.*

Definitions have now been included for the terms interdisciplinary data, interdisciplinary data management.

2. *There seems to be some confusion in the writing about the difference between interdisciplinary collaborations, interdisciplinary studies, and the study of interdisciplinarity.*

These terms have been removed with a stronger emphasis/reference to data produced from interdisciplinary research.

3. *In general, the writing needs to be edited for clarity.*

The writing has been edited for clarity and elements removed/streamlined.

4. *Having read the whole it is not clear, for example, who will have access to the hub – is the intention for collaborators in the project or is it to be an entirely open access resource and if so what are the data protection implications? Will some data be restricted access? Are there charges to access, and what are the plans for sustainability beyond the funded lifespan of the project?*

UKRI demands that all project data after an embargo period is open-access and kept for 10 years. A paragraph has been added on this.

5. *However, the main limitation is that the purpose of this manuscript seems to be to describe the purpose and hoped for outputs of this datahub. This may be an important dissemination task for the project, but an research journal is not the correct home for it. If the authors believe that there is a research question driving this manuscript, it should be set out in the introduction and the remainder of the paper rewritten with this question in mind.*

Confidential: For Review Only

Three underpinning questions are set out in the introduction and detailed across the article. The text has been edited to demonstrate a greater synergy between the introduction and methods. This is an interdisciplinary article with authors representing the fields of computer science, nutrition, clinical medicine and informatics. The article has been refocused on the contribution to new knowledge: the Semantic ontology, decision support tool and data discovery tool.

Confidential: For Review Only

Developing a data repository to support interdisciplinary research into childhood stunting: a UKRI GCRF Action Against Stunting Hub protocol paper

Authors

Kaitlin Conway-Moore[1], Darius Tetsa Tata[2], Peter Wood[3], Val Katerinchuk[3], Dinesh Yadav D M[2], Little Flower Augustine[4], Manne Munikumar[4], Assana Diop[5], Fassiatou Tairou[5], Modou Jobarteh[2], Bharati Kulkarni[4], Babacar Faye[5], Paul Haggarty[6] and Claire Heffernan[2]

Affiliations

[1]Department of Health Services Research and Policy, London School of Hygiene and Tropical Medicine, London, United Kingdom

[2]Department of Population Health, London School of Hygiene and Tropical Medicine, London, United Kingdom

[3]Department of Computer Science and Information Systems, Birkbeck University of London, London, United Kingdom

[4] ICMR-National Institute of Nutrition, Jamai-Osmania PO, Hyderabad, India

[5]Faculté de Médecine, Pharmacie et Odontologie Université Cheikh Anta Diop de Dakar (UCAD), Dakar, Senegal

[6]Rowett Institute of Nutrition and Health, University of Aberdeen, Aberdeen, United Kingdom

Corresponding Author: Claire Heffernan, London International Development Centre, 20 Bloomsbury Square, London, UK WC1A 2NS; email: Claire.Heffernan@lidc.ac.uk

Confidential: For Review Only

Abstract

Introduction

As a topic of inquiry in its own right, data management for interdisciplinary research projects is in its infancy. Key issues include the inability of researchers to effectively query diverse data outputs and to identify potentially important synergies between discipline-specific data. Equally problematic, few semantic ontologies exist to better support data organisation and discovery. Finally, while interdisciplinary research is widely regarded as beneficial to unpacking complex problems, non-researchers such as policymakers and planners often struggle to use and interrogate the related datasets. To address these issues, the following article details the design and development of the UKRI GCRF Action Against Stunting Hub (AASH)'s All-Hub Data Repository (AHDR).

~~Presently, there is little guidance available on the design and implementation of large-scale, interdisciplinary data management systems. To address this, the UKRI GCRF Action Against Stunting Hub (AASH) is creating the All-Hub Data Repository (AHDR), which aims to act as a platform for the management of AASH data. The platform will include an Interdisciplinary Aid to support the analysis of AASH data and a Decision Support Tool relevant for policymakers and planners. The platform will also act as a centralized location for monitoring the AASH's diverse project management activities. Beyond use in our own project, our overarching objective with the AHDR is to create a new roadmap for the management and analysis of interdisciplinary data.~~

Methods and analysis

The AHDR is a single application, single authentication web-based platform comprised of a data warehouse to store data from across the AASH's three study countries and to support data querying. Four novel components of the AHDR are described in the following article: 1) a unique data discovery tool 2) a metadata catalogue that provides researchers with an interface to explore the AASH's data outputs and engage with a new semantic ontology related to child stunting; 3) an interdisciplinary aid to support a directed approach to identifying synergies and interactions between AASH data and 4) a decision support tool that will support non-researchers in engaging with the wider evidence-based outputs of the AASH.

~~The AHDR is a single application, single authentication web-based platform comprised of five interlinked elements: 1) a data warehouse to store data from across the AASH's three study countries and to support data querying; 2) a metadata catalogue that provides researchers with an interface to explore the AASH's data outputs and engage with a new interdisciplinary ontology related to child stunting; 3) an Interdisciplinary Aid that supports a directed approach to identifying synergies and interactions between the drivers stunting 4) a set of Tableau dashboards that enable AASH project staff to track progress towards monitoring, evaluation and learning goals; and 5) a Decision Support Tool that will utilize AASH data to engage with the wider evidence-based outputs of the AASH.~~

Ethics and dissemination

Confidential: For Review Only

Ethical approval for this study was granted by institutional ethics committees in the United Kingdom, India, Indonesia, and Senegal. Results will be disseminated via publications in peer-reviewed journals; presentations at international conferences and community-level public engagement events; key stakeholder meetings; and in public repositories with appropriate Creative Commons licences allowing for the widest possible use.

Summary Box

What is known about the subject

- Data management for large-scale interdisciplinary research projects can pose significant challenge to researchers.

What this study hopes to add

- Insight into designing a unique data management system, with recommendations for developing an integrated structure capable of supporting interdisciplinary analysis, storage, querying and decision support.

How this study might affect research, practice or policy

- By identifying a range of data management innovations through our own large-scale interdisciplinary research project, this work has the potential to inform the field.

Confidential: For Review Only

Introduction

Interdisciplinary research centres on the creation of new knowledge from the explicit combination of different disciplines, sub-disciplines or topics, with interdisciplinary research and the underpinning collaboration recognised to be better at unpacking complex problems and supporting innovative solutions. Despite growing attention in recent years to the merits of interdisciplinary research, however, tools for effective data management remain limited.(1,2) As a result, interdisciplinary data analysis is often done in an ad hoc manner, with researchers often left to their own devices. (3,4) Relatedly, there is little guidance in the literature on how to manage data from multiple disciplines to identify the most promising synergies and inter-relationships between such data. To address these gaps, researchers are moving to develop semantic ontologies or agreed vocabularies to search and organise data around particular topics.

While the development of semantic ontologies clearly enhances the ability of researchers to explore and analyse data across multiple disciplines within a particular field, the needs of other stakeholders of these research outputs are less well addressed. In particular, the ability of policymakers and planners to mine interdisciplinary datasets is limited. For example, there are currently a handful of widely-used global datasets related to child nutrition (e.g. WHO Global Database on Child Growth and Malnutrition, the World Bank Health Nutrition and Population Statistics, the UNICEF-WHO-World Bank Joint Child Malnutrition Estimates or indeed, the University of Oxford's Young Lives dataset), yet these tools do not explicitly focus on cross-disciplinary synergies within their data outputs.

Seeking to address these issues and inform the emerging field of interdisciplinary data management, the following article details the protocol for developing the UKRI GCRF Action Against Stunting Hub (AASH)'s All-Hub Data Repository (AHDR). The Hub's 18 institutional

Confidential: For Review Only

collaborators will collect and analyse approximately 800,000 data points from study participants in India, Indonesia and Senegal. As such, we are collecting a diverse array of data from surveys, direct observations and anthropometric measurements to focus group discussions and in-depth qualitative interviews to biological and environmental samples.

The AHDR is underpinned by three key questions:

1. What is the best platform for the storage and querying of a diverse three-country comparative dataset related to child stunting?
2. What does a new semantic ontology for child nutritional research look like?
3. Can such a data management tool also be used to create a decision-support tool relevant for diverse audiences of policymakers and planners working on child stunting globally?

A key aim of the AHDR will be to make anonymized AASH data public facing as part of the UKRI's open research data strategy, which seeks to make research processes and findings as transparent, understandable, and reproducible as possible. While access to AASH data will be restricted to project staff during the project and initial data analysis period (18-months from the project end-date), at the end of this period, all restrictions on data access will be removed and data licences updated to enable free download. In line with UKRI guidelines, this data will be accessible for 10 years after the project ends, and held on a London School of Hygiene &Tropical Medicine server for this period.

MEL dashboards will be used to assess whether study activities are proceeding in line with the project timeline, thus allowing our project management teams in each country to view progress in real time and identify bottlenecks. In order to ensure our MEL dashboards are up-to-date, we have created links between our project management and communication software and the dashboards themselves.

Fourth, a critical component of the AHDR is a decision support tool for policymakers, practitioners and field staff. The goal of this tool is to predict a child's risk for being on the pathway to stunting. Using AASH data to create a predictive model, a traffic light system will be created to identify children at different levels of risk. By collating the results at the geographic level, a range of stakeholders from health workers to policy makers and planners can then better tailor/focus programmes for maximum impact.

A key aim of the AHDR will be to make anonymized AASH data public facing as part of the UKRI's open research data strategy, which seeks to make research processes and findings as transparent, understandable, and reproducible as possible. While access to AASH data will be restricted to project staff during the implementation phase of the project and an initial data analysis period, at the end of this period, all restrictions on data access will be removed and data licences updated to enable free download.

To this end, the aforementioned AHDR components will be integrated into a single publicly accessible web application with a landing page that presents and describes how to navigate through each of the AHDR components. The landing page will provide direct access to each of the components with a centralised authentication mechanism, hence reducing complexity and enhancing security. Application Programming Interfaces (APIs) will be used to digest information between the various components and external resources. The AHDR will be hosted and maintained on the London School of Hygiene and Tropical Medicine's IT infrastructure for a

Confidential: For Review Only

minimum of 10 years. A Data Management Committee (DMC) has also been created to oversee and ensure the long-term planning and maintenance of the AHDR.

Methods and analysis

All-Hub Data Repository Design

The Data Warehouse, the Data Download Tool and the Interdisciplinary Aid

The AHDR data warehouse will integrate and store AASH data from the various field-level data collection tools, including CommCare, KoboCollect, REDCap and laboratory analyses using an automated Integration Platform as a Service (iPaaS) middleware system, powered by OpenFn.(5–8)(5–8)The iPaaS middleware will either link to these data collection platforms directly via API connections, or to Secure File Transfer Protocol (SFTP) servers that will contain ready-to-export data in CSV or XLS formats. To ensure that there is alignment in the types of data collected across our three study countries, all metadata from our data collection applications have undergone an extensive process of labelling, mapping and review.

To prepare data stored in the data warehouse for analysis by end users, measures will be put in place for automated data cleansing both at the point of collection and during the data integration process. Specifically, data validation rules and skip logic will be implemented in different Clinical Data Management Applications (CDMAs) to reduce the risk of erroneous and inconsistent data. In addition to this, external scripts will be written in R, STATA and Python to enforce data quality checks in the event they cannot be implemented into the CDMAs, and for the purposes of anonymising/pseudo-anonymising data prior to transfer into the data warehouse. Data integration programs will also be designed to map data from across the AASH by harmonising common variable names and labels; standardising values; calculating derived variables; and recoding data as required. Data collection and transformation will be done in accordance with AASH study protocols, standard operating procedures (SOPs), General Data Protection Regulation (GDPR) guidelines and both national and local regulations for data protection in Senegal, India and Indonesia.

The data warehouse has been built using PostgreSQL, an open-source database management system that specializes in securely storing complex datasets.(9) Genomic data collected as part of our study will be held on the European Geno-phenome Archive (EGA) and connected to the AHDR using APIs. Each of our three project countries will also be equipped with high-performance computing (HPC) servers that allow for the maintenance, computing and analysis of these large files. Researchers seeking access to genomic data will be able to do so via the AHDR, following completion of consent and authorisation forms, as required. The AHDR's data warehouse will also store a limited set of derived and summary variables related to genomic data.

FIGURE 1 HERE

Figure 1: Data Download Tool for the Data Warehousediscovery tool for the data warehouse

Confidential: For Review Only

A Data Discovery Tool, seen in **Figure 1**, is being implemented to enable researchers and other end users to browse and download data from the data warehouse. This functionality will enable both viewing and downloading in raw CSV file format and a CSV download displaying metadata labels in place of encoded column names and multi-choice values. To facilitate the cross-disciplinary analysis, it will be possible to navigate through individual columns of the various data tables and combine them into a single CSV output, joined on a unique subject identification number, or alternative identifiers when a subject identification number is not present.

The Interdisciplinary Aid (IA) will be created by linking the various sources of data collected across our project sites to both topic-based and conceptual entities through a Neo4j graph database system (Version 4). All data variables will be tagged and allocated to a position within a defined hierarchy using specific domain ontologies. The IA will enable the establishment of direct connections between variables based on their syntactic matching with data tags and/or within the hierarchy. The tool will also enable the establishment of indirect connections between variables and datasets based on their semantic proximity (i.e., ontological connection) with the aid of semantic matching algorithms. Researchers will then have a visual interface that they can browse to narrow in on the types of data they find most useful to their work, while also finding conceptual/logical connections to data outside their primary discipline. Finally, research collaboration will also be supported via the platform's use of CKAN (Version 2.9), an open-source data portal. With CKAN, researchers will be able to upload analysed and partially analysed datasets for use by other researchers both within and outside of their discipline. In doing so, the building blocks of interdisciplinary analysis will be made available to all end users.

A Data Download Tool, seen in **Figure 1**, is being implemented to enable researchers to browse and download data from the data warehouse. This functionality will enable both viewing and downloading in raw CSV file format and a CSV download displaying metadata labels in place of encoded column names and multi-choice values. Alternatively, the metadata may be downloaded and viewed separately for any selection of data. To facilitate interdisciplinary research, it will be possible to navigate through individual columns of the various data tables and combine them into a single CSV output, joined on a unique subject identification number, or alternative identifiers when a subject identification number is not present.

The Interdisciplinary Aid will be created by linking the various sources of data collected across our project sites to both topic-based and conceptual entities through a Neo4j graph database system (Version 4). All data variables will be tagged and allocated to a position within a defined hierarchy using specific domain ontologies.

The Interdisciplinary Aid will enable the establishment of direct connections between variables based on their syntactic matching with data tags and/or within the hierarchy. The tool will also enable the establishment of indirect connections between variables and datasets based on their semantic proximity (i.e., ontological connection) with the aid of semantic matching algorithms. Researchers will then have a visual interface that they can browse to narrow in on the types of data they find most useful to their work, while also finding conceptual/logical connections to data outside their primary discipline. Finally, interdisciplinarity will also be supported via the platform's use of CKAN (Version 2.9), an open-source data portal. With CKAN, researchers will be able to upload analysed and partially analysed datasets for use by other researchers both

Confidential: For Review Only

within and outside of their discipline. In doing so, the building blocks of interdisciplinary analysis will be made available to all researchers.

Metadata Catalogue/Semantic Ontology

The AHDR metadata catalogue will provide an overview of all the AASH data available for download and analysis. Underpinning the metadata catalogue will be a new semantic ontology for child stunting. To create the semantic ontology, first, terms from existing ontologies from across the thematic disciplines involved in the AASH (e.g., MeSH, FoodOn, ECCD Ontology) will be mined.(10,11) Second, expert consultations will take place to check, define and agree terms. The terms will then be used to organize the different classes of data collected by the AASH into a hierarchy based on their various categories, the types of relationships between them, and their potential 'nodes' or areas where disciplines overlap.(12,13)

Drawing on monitoring forms and reporting routinely submitted by in-country project staff, the iPaaS middleware will extract relevant data, summarize it, and calculate key indicators. These data extractions and summaries will then be stored in the data warehouse, where they will serve as the data source for a series of country and work package-specific Tableau dashboards used to visualize progress and provide real-time updates on all project activities. The Tableau dashboards will be password protected, and organized by data access groups and via access roles. A visualization of this Glasscubes-iPaas-AHDR-Tableau data integration process can be seen in **Figure 2**.

FIGURE 2 HERE

Figure 2: AHDR Data Integration for MEL

The Decision Support Tool

The AHDR aims to provide a decision support tool to enable policymakers, practitioners and field staff to interrogate AASH data. The tool will predict a child's risk for being on the pathway to stunting. The app will prompt users for a number of input variables which will be sent back to the Python model to predict a traffic light colour. Additionally bulk-processing functionality will be available via uploading a file of input variables. Users can then download their file, modified with a predicted colour for each row of input variables in the input file. Various visualisations, including geographic visualisation, based on the bulk data, will also be provided.

As one of the final outputs of our work, the AHDR aims to provide a decision support tool for policymakers, practitioners and field staff. As noted above, the goal of this tool is to predict a child's risk for being on the pathway to stunting. The prediction system will be based on a statistical model. The app will prompt users for a number of input variables which will be sent back to the Python model to predict a traffic light colour. Additionally bulk-processing functionality will be available via uploading a file of input variables. Users can then download their file, modified with a predicted colour for each row of input variables in the input file. Various visualisations, including geographic visualisation, based on the bulk data will also be provided.

Confidential: For Review Only

Figure 2~~3~~ provides an overview of the interconnected components of the AHDR as outlined above, including the ways in which it integrates inputs held in our project management and communication application (i.e., Glasscubes), with our field-data collection software (i.e., CommCare, Kobo Connect, and REDCAP), and genomic data stored on high performance computers. As seen below, all data from these sources will be integrated via our iPaaS middleware and sent to our data warehouse. The data warehouse will then feed all structured data into the web-based data portal, where researchers and other key stakeholders can interact with and export AASH data chosen from both thematic and interdisciplinary combinations. Interested parties will ultimately also be able to draw information from the data warehouse using the interdisciplinary aid and decision support tool, and our project management staff will have secure access to information related to our project's progress via the MEL Tableau dashboards.

FIGURE 2~~3~~ HERE

Figure 2~~3~~: Visualisation of the All-Hub Data Repository (AHDR)

Patient and public involvement

Participant and public involvement towards the eradication of child stunting is at the core of our project. We are working directly with the local communities in our study countries in India, Indonesia and Senegal, respectively, to understand how stunting and the work of the AASH has impacted their daily lives. Along with this, with a strong focus on user experience and interaction at the core of the AHDR's design, we aim for our work to bring researchers from different disciplines together to share knowledge and expertise, including allowing researchers to upload their analyzed datasets for use by others. The AHDR also aims to provide a resource for policymakers and practitioners to hone interventions that will have the highest possible benefit to the general public.

Ethics and dissemination

Ethical approval for the Action Against Stunting Hub, inclusive of the All-Hub Data Repository, was granted by the Ethics Committee of the London School of Hygiene and Tropical Medicine (17915/RR/17513); the Social Science Research Ethical Review Board at the Royal Veterinary College (URN SR2020-0197); and the International Livestock Research Institute Institutional Research Ethics Committee (ILRI-IREC2020-33). Research site-specific approvals were granted by the National Institute of Nutrition (ICMR), Ministry of Health and Family Welfare, Government of India (CR/04/I/2021); Health Research Ethics Committee, University of Indonesia and Cipto Mangunkusumo Hospital (KET-887/UN2.F1/ETIK/PPM.00.02/2019); and the Comité National d'Ethique pour la Recherche en Santé, Senegal (Protocole SEN19/78).

Dissemination will include publications in international peer-reviewed journals; community-level public engagement events, national and international conferences and key stakeholder meetings; and the use of the AHDR by our vast network of AASH researchers, and ultimately, the wider research and policymaking community. The AASH will seek to make data as open as possible as is consistent with UKRI priorities and FAIR (Findable, Accessible, Interoperable, Reusable) data principles. Upon publication, study data will be posted in public repositories

Confidential: For Review Only

(preferably domain-specific), or as required by the relevant publication body. Published data will be made available with a preference for minimal licence restrictions (typically CC BY, CC BY-NC, or CC BY-ND) as per UKRI guidance, following assessment for exploitation potential.

Authors' contributions

KCM and CH drafted the manuscript; KCM, CH, DTT, PW and VK provided critical revision to the manuscript; CH, PW, VK and DTT designed the tool, KCM, DTT, PW, VK, DYDM, LFA, MM, AD, and FT contributed to the design. All authors read and approved the final manuscript. We acknowledge and thank Taylor Downs, Aleksa Krolls, Joseph Sam and Aissatou Diallo at OpenFn who the Action Against Stunting Hub contracted to build the AHDR.

Funding statement: This work is supported by UKRI-GCRF Action Against Stunting Hub, grant number MR/S01313X/1.

Competing interests statement: The authors declare no competing interests.

References

1. Gohar F, Maschmeyer P, Mfarrej B, Lemaire M, Wedderburn LR, Roncarolo MG, et al. Driving Medical Innovation Through Interdisciplinarity: Unique Opportunities and Challenges. Front Med (Lausanne). 2019;6(35):1–6.
2. Rey J, Koplan P, Bond C, Merson MH, Reddy S, Rodriguez MH, et al. Towards a common definition of global health. The Lancet. 2009;373:1993–5.
3. Tobi H, Kampen JK. Research design: the methodology for interdisciplinary research framework. Qual Quant. 2018 May 1;52(3):1209–25.
4. Heffernan C. Building synergies in development research: Is it time for the mosaic approach? Dev Pract. 2008 Nov;18(6):686–700.
5. Dimagi. CommCare [Internet]. 2022 [cited 2022 Aug 1]. Available from: https://www.dimagi.com/commcare/
6. Kobo Inc. About KoBo [Internet]. 2022 [cited 2022 Sep 9]. Available from: https://www.kobotoolbox.org/kobo/
7. Vanderbilt. About REDCap [Internet]. 2022 [cited 2022 Sep 9]. Available from: https://projectredcap.org/about/
8. OpenFn. Automation & Data Integration [Internet]. 2022 [cited 2022 Apr 8]. Available from: https://www.openfn.org/
9. The PostgreSQL Global Development Group. What is PostgreSQL? [Internet]. 2022 [cited 2022 Sep 9]. Available from: https://www.postgresql.org/about/
10. Maedche A, Staab S. Ontology Learning for the Semantic Web. IEEE Intell Syst. 2001;16(2):72–9.

Confidential: For Review Only

11. Noy NF. Semantic integration: A survey of ontology-based approaches. SIGMOD Rec. 2004;33(4):65–70.

12. Ontotext. What is a Knowledge Graph? [Internet]. 2022 [cited 2022 Nov 2]. Available from: https://www.ontotext.com/knowledgehub/fundamentals/what-is-a-knowledge-graph/

13. Jacob EK. Ontologies and the Semantic Web. Bulletin of the American Society for Information Science and Technology. 2003 Apr 1;29(4):19–22.

Confidential: For Review Only

BMJ Paediatrics Open

Developing a data repository to support interdisciplinary research into childhood stunting: a UKRI GCRF Action Against Stunting Hub protocol paper

Journal:	*BMJ Paediatrics Open*
Manuscript ID	bmjpo-2023-002443.R1
Article Type:	Protocol
Date Submitted by the Author:	16-Apr-2024
Complete List of Authors:	Conway-Moore, Kaitlin; London School of Hygiene & Tropical Medicine, Health Services Research and Policy<br>Tata, Darius; London International Development Centre (LIDC), London, UK<br>Wood, Peter; Birkbeck University of London<br>Katerinchuk, Val; Birkbeck University of London<br>Yadav, Dinesh; London School of Hygiene & Tropical Medicine, Department of Population Health<br>Augustine, Little Flower; ICMR-National Institute of Nutrition<br>Munikumar, Manne; ICMR-National Institute of Nutrition,<br>Diop, Assana; Service de Parasitologie-Mycologie- Pédiatrie, Faculté de médecine, Université Cheikh Anta Diop (UCAD), Dakar, Senegal<br>Tairou, Fassiatou; Service de Parasitologie-Mycologie- Pédiatrie, Faculté de médecine, Université Cheikh Anta Diop (UCAD), Dakar, Senegal<br>Jobarteh, Modou; London School of Hygiene & Tropical Medicine, Epidemiology & Population Health<br>Kulkarni, Bharati; National Institute of Nutrition<br>Faye, Babacar; Université Cheikh Anta Diop (UCAD)<br>Haggarty, Paul; University of Aberdeen<br>Heffernan, Claire; London School of Hygiene and Tropical Medicine, Department of Population Health
Keywords:	Data Collection, Growth, Physiology

SCHOLARONE™
Manuscripts

I, the Submitting Author has the right to grant and does grant on behalf of all authors of the Work (as defined in the below author licence), an exclusive licence and/or a non-exclusive licence for contributions from authors who are: i) UK Crown employees; ii) where BMJ has agreed a CC-BY licence shall apply, and/or iii) in accordance with the terms applicable for US Federal Government officers or employees acting as part of their official duties; on a worldwide, perpetual, irrevocable, royalty-free basis to BMJ Publishing Group Ltd ("BMJ") its licensees and where the relevant Journal is co-owned by BMJ to the co-owners of the Journal, to publish the Work in this journal and any other BMJ products and to exploit all rights, as set out in our licence.

The Submitting Author accepts and understands that any supply made under these terms is made by BMJ to the Submitting Author unless you are acting as an employee on behalf of your employer or a postgraduate student of an affiliated institution which is paying any applicable article publishing charge ("APC") for Open Access articles. Where the Submitting Author wishes to make the Work available on an Open Access basis (and intends to pay the relevant APC), the terms of reuse of such Open Access shall be governed by a Creative Commons licence – details of these licences and which Creative Commons licence will apply to this Work are set out in our licence referred to above.

Other than as permitted in any relevant BMJ Author's Self Archiving Policies, I confirm this Work has not been accepted for publication elsewhere, is not being considered for publication elsewhere and does not duplicate material already published. I confirm all authors consent to publication of this Work and authorise the granting of this licence.

Confidential: For Review Only

Developing a data repository to support interdisciplinary research into childhood stunting: a UKRI GCRF Action Against Stunting Hub protocol paper

Authors

Kaitlin Conway-Moore[1], Darius Tetsa Tata[2], Peter Wood[3], Val Katerinchuk[3], Dinesh Yadav D M[2], Little Flower Augustine[4], Manne Munikumar[4], Assana Diop[5], Fassiatou Tairou[5], Modou Lamin Jobarteh[2], Bharati Kulkarni[4], Babacar Faye[5], Paul Haggarty[6] and Claire Heffernan[2]

Affiliations

[1]Department of Health Services Research and Policy, London School of Hygiene and Tropical Medicine, London, United Kingdom

[2]Department of Population Health, London School of Hygiene and Tropical Medicine, London, United Kingdom

[3]Department of Computer Science and Information Systems, Birkbeck University of London, London, United Kingdom

[4] ICMR-National Institute of Nutrition, Jamai-Osmania PO, Hyderabad, India

[5]Faculté de Médecine, Pharmacie et Odontologie Université Cheikh Anta Diop de Dakar (UCAD), Dakar, Senegal

[6]Rowett Institute of Nutrition and Health, University of Aberdeen, Aberdeen, United Kingdom

Corresponding Author: Claire Heffernan, London International Development Centre, 20 Bloomsbury Square, London, UK WC1A 2NS; email: Claire.Heffernan@lidc.ac.uk

Confidential: For Review Only

Abstract

Introduction

As a topic of inquiry in its own right, data management for interdisciplinary research projects is in its infancy. Key issues include the inability of researchers to effectively query diverse data outputs and to identify potentially important synergies between discipline-specific data. Equally problematic, few semantic ontologies exist to better support data organisation and discovery. Finally, while interdisciplinary research is widely regarded as beneficial to unpacking complex problems, non-researchers such as policymakers and planners often struggle to use and interrogate the related datasets. To address these issues, the following article details the design and development of the UKRI GCRF Action Against Stunting Hub (AASH)'s All-Hub Data Repository (AHDR).

Methods and analysis

The AHDR is a single application, single authentication web-based platform comprised of a data warehouse to store data from across the AASH's three study countries and to support data querying. Four novel components of the AHDR are described in the following article: 1) a unique data discovery tool 2) a metadata catalogue that provides researchers with an interface to explore the AASH's data outputs and engage with a new semantic ontology related to child stunting; 3) an interdisciplinary aid to support a directed approach to identifying synergies and interactions between AASH data and 4) a decision support tool that will support non-researchers in engaging with the wider evidence-based outputs of the AASH.

Ethics and dissemination

Ethical approval for this study was granted by institutional ethics committees in the United Kingdom, India, Indonesia, and Senegal. Results will be disseminated via publications in peer-reviewed journals; presentations at international conferences and community-level public engagement events; key stakeholder meetings; and in public repositories with appropriate Creative Commons licences allowing for the widest possible use.

Confidential: For Review

What is known about the subject

- Data management for large-scale interdisciplinary research projects can pose significant challenge to researchers.

What this study hopes to add

- Insight into designing a unique data management system, with recommendations for developing an integrated structure capable of supporting interdisciplinary analysis, storage, querying and decision support.

How this study might affect research, practice or policy

- By identifying a range of data management innovations through our own large-scale interdisciplinary research project, this work has the potential to inform the field.

Introduction

Interdisciplinary research centres on the creation of new knowledge from the explicit combination of different disciplines, sub-disciplines or topics, with interdisciplinary research and the underpinning collaboration recognised to be better at unpacking complex problems and supporting innovative solutions. Despite growing attention in recent years to the merits of interdisciplinary research, however, tools for effective data management remain limited.(1,2) As a result, interdisciplinary data analysis is often done in an ad hoc manner, with researchers often left to their own devices. (3,4) Relatedly, there is little guidance in the literature on how to manage data from multiple disciplines to identify the most promising synergies and inter-relationships between such data. To address these gaps, researchers are moving to develop semantic ontologies or agreed vocabularies to search and organise data around particular topics.

While the development of semantic ontologies clearly enhances the ability of researchers to explore and analyse data across multiple disciplines within a particular field, the needs of other stakeholders of these research outputs are less well addressed. In particular, the ability of policymakers and planners to mine interdisciplinary datasets is limited. For example, there are currently a handful of widely-used global datasets related to child nutrition (e.g. WHO Global Database on Child Growth and Malnutrition, the World Bank Health Nutrition and Population Statistics, the UNICEF-WHO-World Bank Joint Child Malnutrition Estimates or indeed, the University of Oxford's Young Lives dataset), yet these tools do not explicitly focus on cross-disciplinary synergies within their data outputs.

Seeking to address these issues and inform the emerging field of interdisciplinary data management, the following article details the protocol for developing the UKRI GCRF Action Against Stunting Hub (AASH)'s All-Hub Data Repository (AHDR). The Hub's 18 institutional collaborators will collect and analyse approximately 800,000 data points from study participants in India, Indonesia and Senegal. As such, we are collecting a diverse array of data from surveys, direct observations and anthropometric measurements to focus group discussions and in-depth qualitative interviews to biological and environmental samples.

The AHDR is underpinned by three key questions:

1. What is the best platform for the storage and querying of a diverse three-country comparative dataset related to child stunting?
2. What does a new semantic ontology for child nutritional research look like?
3. Can such a data management tool also be used to create a decision-support tool relevant for diverse audiences of policymakers and planners working on child stunting globally?

A key aim of the AHDR will be to make anonymized AASH data public facing as part of the UKRI's open research data strategy, which seeks to make research processes and findings as transparent, understandable, and reproducible as possible. While access to AASH data will be restricted to project staff during the project and initial data analysis period (18-months from the project end-date), at the end of this period, all restrictions on data access will be removed and data licences updated to enable free download. In line with UKRI guidelines, this data will be

Confidential: For Review Only

accessible for 10 years after the project ends, and held on a London School of Hygiene &Tropical Medicine server for this period.

Methods and analysis

All-Hub Data Repository Design

The Data Warehouse, the Data Discovery Tool and the Interdisciplinary Aid

The AHDR data warehouse will integrate and store AASH data from the various field-level data collection tools, including CommCare, KoboCollect, REDCap and laboratory analyses using an automated Integration Platform as a Service (iPaaS) middleware system, powered by OpenFn.(5–8) The iPaaS middleware will either link to these data collection platforms directly via API connections, or to Secure File Transfer Protocol (SFTP) servers that will contain ready-to-export data in CSV or XLS formats. To ensure that there is alignment in the types of data collected across our three study countries, all metadata from our data collection applications have undergone an extensive process of labelling, mapping and review.

To prepare data stored in the data warehouse for analysis by end users, measures will be put in place for automated data cleansing both at the point of collection and during the data integration process. Specifically, data validation rules and skip logic will be implemented in different Clinical Data Management Applications (CDMAs) to reduce the risk of erroneous and inconsistent data. In addition to this, external scripts will be written in R, STATA and Python to enforce data quality checks in the event they cannot be implemented into the CDMAs, and for the purposes of anonymising/pseudo-anonymising data prior to transfer into the data warehouse. Data integration programs will also be designed to map data from across the AASH by harmonising common variable names and labels; standardising values; calculating derived variables; and recoding data as required. Data collection and transformation will be done in accordance with AASH study protocols, standard operating procedures (SOPs), General Data Protection Regulation (GDPR) guidelines and both national and local regulations for data protection in Senegal, India and Indonesia.

The data warehouse has been built using PostgreSQL, an open-source database management system that specializes in securely storing complex datasets.(9) Genomic data collected as part of our study will be held on the European Geno-phenome Archive (EGA) and connected to the AHDR using APIs. Each of our three project countries will also be equipped with high-performance computing (HPC) servers that allow for the maintenance, computing and analysis of these large files. Researchers seeking access to genomic data will be able to do so via the AHDR, following completion of consent and authorisation forms, as required. The AHDR's data warehouse will also store a limited set of derived and summary variables related to genomic data.

FIGURE 1 HERE

Figure 1: Data discovery tool for the data warehouse

Confidential: For Review Only

A Data Discovery Tool, seen in **Figure 1**, is being implemented to enable researchers and other end users to browse and download data from the data warehouse. This functionality will enable both viewing and downloading in raw CSV file format and a CSV download displaying metadata labels in place of encoded column names and multi-choice values. To facilitate the cross-disciplinary analysis, it will be possible to navigate through individual columns of the various data tables and combine them into a single CSV output, joined on a unique subject identification number, or alternative identifiers when a subject identification number is not present.

The Interdisciplinary Aid (IA) will be created by linking the various sources of data collected across our project sites to both topic-based and conceptual entities through a Neo4j graph database system (Version 4). All data variables will be tagged and allocated to a position within a defined hierarchy using specific domain ontologies. The IA will enable the establishment of direct connections between variables based on their syntactic matching with data tags and/or within the hierarchy. The tool will also enable the establishment of indirect connections between variables and datasets based on their semantic proximity (i.e., ontological connection) with the aid of semantic matching algorithms. Researchers will then have a visual interface that they can browse to narrow in on the types of data they find most useful to their work, while also finding conceptual/logical connections to data outside their primary discipline. Finally, research collaboration will also be supported via the platform's use of CKAN (Version 2.9), an open-source data portal. With CKAN, researchers will be able to upload analysed and partially analysed datasets for use by other researchers both within and outside of their discipline. In doing so, the building blocks of interdisciplinary analysis will be made available to all end users.

Meta-Data Catalogue/Semantic Ontology

The AHDR metadata catalogue will provide an overview of all the AASH data available for download and analysis. Underpinning the metadata catalogue will be a new semantic ontology for child stunting. To create the semantic ontology, first, terms from existing ontologies from across the thematic disciplines involved in the AASH (e.g., MeSH, FoodOn, ECCD Ontology) will be mined.(10,11) Second, expert consultations will take place to check, define and agree terms. The terms will then be used to organize the different classes of data collected by the AASH into a hierarchy based on their various categories, the types of relationships between them, and their potential 'nodes' or areas where disciplines overlap.(12,13)

The Decision Support Tool

The AHDR aims to provide a decision support tool to enable policymakers, practitioners and field staff to interrogate AASH data. The tool will predict a child's risk for being on the pathway to stunting. The app will prompt users for a number of input variables which will be sent back to the Python model to predict a traffic light colour. Additionally bulk-processing functionality will be available via uploading a file of input variables. Users can then download their file, modified with a predicted colour for each row of input variables in the input file. Various visualisations, including geographic visualisation, based on the bulk data, will also be provided.

Figure 2 provides an overview of the interconnected components of the AHDR as outlined above, including the ways in which it integrates inputs held in our project management and

Confidential: For Review Only

communication application (i.e., Glasscubes), with our field-data collection software (i.e., CommCare, Kobo Connect, and REDCAP), and genomic data stored on high performance computers. As seen below, all data from these sources will be integrated via our iPaaS middleware and sent to our data warehouse. The data warehouse will then feed all structured data into the web-based data portal, where researchers and other key stakeholders can interact with and export AASH data chosen from both thematic and interdisciplinary combinations. Interested parties will ultimately also be able to draw information from the data warehouse using the interdisciplinary aid and decision support tool, and our project management staff will have secure access to information related to our project's progress via the MEL Tableau dashboards.

FIGURE 2 HERE

Figure 2: Visualisation of the All-Hub Data Repository (AHDR)

Patient and public involvement

Participant and public involvement towards the eradication of child stunting is at the core of our project. We are working directly with the local communities in our study countries in India, Indonesia and Senegal, respectively, to understand how stunting and the work of the AASH has impacted their daily lives. Along with this, with a strong focus on user experience and interaction at the core of the AHDR's design, we aim for our work to bring researchers from different disciplines together to share knowledge and expertise, including allowing researchers to upload their analyzed datasets for use by others. The AHDR also aims to provide a resource for policymakers and practitioners to hone interventions that will have the highest possible benefit to the general public.

Ethics and dissemination

Ethical approval for the Action Against Stunting Hub, inclusive of the All-Hub Data Repository, was granted by the Ethics Committee of the London School of Hygiene and Tropical Medicine (17915/RR/17513); the Social Science Research Ethical Review Board at the Royal Veterinary College (URN SR2020-0197); and the International Livestock Research Institute Institutional Research Ethics Committee (ILRI-IREC2020-33). Research site-specific approvals were granted by the National Institute of Nutrition (ICMR), Ministry of Health and Family Welfare, Government of India (CR/04/I/2021); Health Research Ethics Committee, University of Indonesia and Cipto Mangunkusumo Hospital (KET-887/UN2.F1/ETIK/PPM.00.02/2019); and the Comité National d'Ethique pour la Recherche en Santé, Senegal (Protocole SEN19/78).

Dissemination will include publications in international peer-reviewed journals; community-level public engagement events, national and international conferences and key stakeholder meetings; and the use of the AHDR by our vast network of AASH researchers, and ultimately, the wider research and policymaking community. The AASH will seek to make data as open as possible as is consistent with UKRI priorities and FAIR (Findable, Accessible, Interoperable, Reusable) data principles. Upon publication, study data will be posted in public repositories (preferably domain-specific), or as required by the relevant publication body. Published data will

Confidential: For Review Only

be made available with a preference for minimal licence restrictions (typically CC BY, CC BY-NC, or CC BY-ND) as per UKRI guidance, following assessment for exploitation potential.

Authors' contributions

KCM and CH drafted the manuscript; KCM, CH, DTT, PW and VK provided critical revision to the manuscript; CH, PW, VK and DTT designed the tool, KCM, DTT, PW, VK, DYDM, LFA, MM, AD, and FT contributed to the design. All authors read and approved the final manuscript. We acknowledge and thank Taylor Downs, Aleksa Krolls, Joseph Sam and Aissatou Diallo at OpenFn who the Action Against Stunting Hub contracted to build the AHDR.

Funding statement: This work is supported by UKRI-GCRF Action Against Stunting Hub, grant number MR/S01313X/1.

Competing interests statement: The authors declare no competing interests.

References

1. Gohar F, Maschmeyer P, Mfarrej B, Lemaire M, Wedderburn LR, Roncarolo MG, et al. Driving Medical Innovation Through Interdisciplinarity: Unique Opportunities and Challenges. Front Med (Lausanne). 2019;6(35):1–6.
2. Rey J, Koplan P, Bond C, Merson MH, Reddy S, Rodriguez MH, et al. Towards a common definition of global health. The Lancet. 2009;373:1993–5.
3. Tobi H, Kampen JK. Research design: the methodology for interdisciplinary research framework. Qual Quant. 2018 May 1;52(3):1209–25.
4. Heffernan C. Building synergies in development research: Is it time for the mosaic approach? Dev Pract. 2008 Nov;18(6):686–700.
5. Dimagi. CommCare [Internet]. 2022 [cited 2022 Aug 1]. Available from: https://www.dimagi.com/commcare/
6. Kobo Inc. About KoBo [Internet]. 2022 [cited 2022 Sep 9]. Available from: https://www.kobotoolbox.org/kobo/
7. Vanderbilt. About REDCap [Internet]. 2022 [cited 2022 Sep 9]. Available from: https://projectredcap.org/about/
8. OpenFn. Automation & Data Integration [Internet]. 2022 [cited 2022 Apr 8]. Available from: https://www.openfn.org/
9. The PostgreSQL Global Development Group. What is PostgreSQL? [Internet]. 2022 [cited 2022 Sep 9]. Available from: https://www.postgresql.org/about/
10. Maedche A, Staab S. Ontology Learning for the Semantic Web. IEEE Intell Syst. 2001;16(2):72–9.
11. Noy NF. Semantic integration: A survey of ontology-based approaches. SIGMOD Rec. 2004;33(4):65–70.
12. Ontotext. What is a Knowledge Graph? [Internet]. 2022 [cited 2022 Nov 2]. Available from: https://www.ontotext.com/knowledgehub/fundamentals/what-is-a-knowledge-graph/

Confidential: For Review Only

13. Jacob EK. Ontologies and the Semantic Web. Bulletin of the American Society for Information Science and Technology. 2003 Apr 1;29(4):19–22.

Confidential: For Review Only

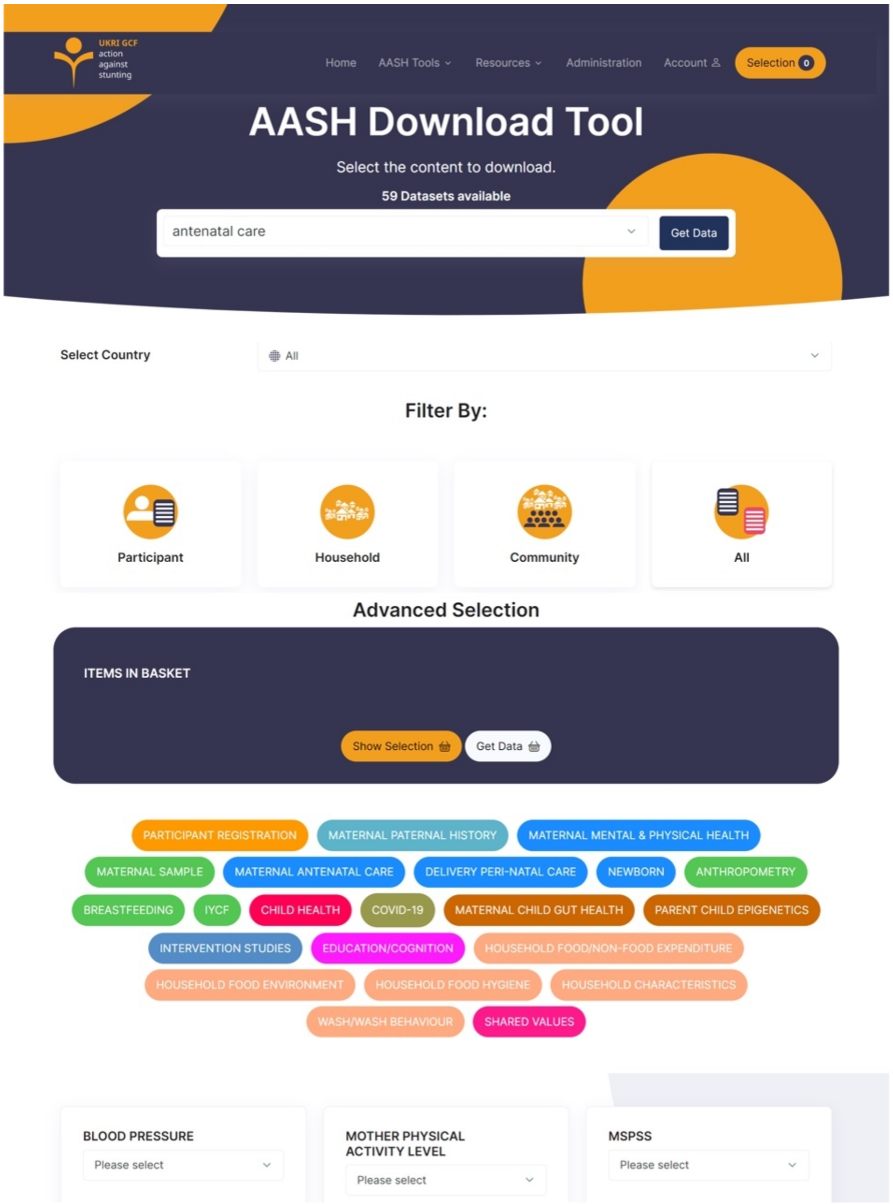

Figure 1: Data discovery tool for the data warehouse

110x148mm (220 x 220 DPI)

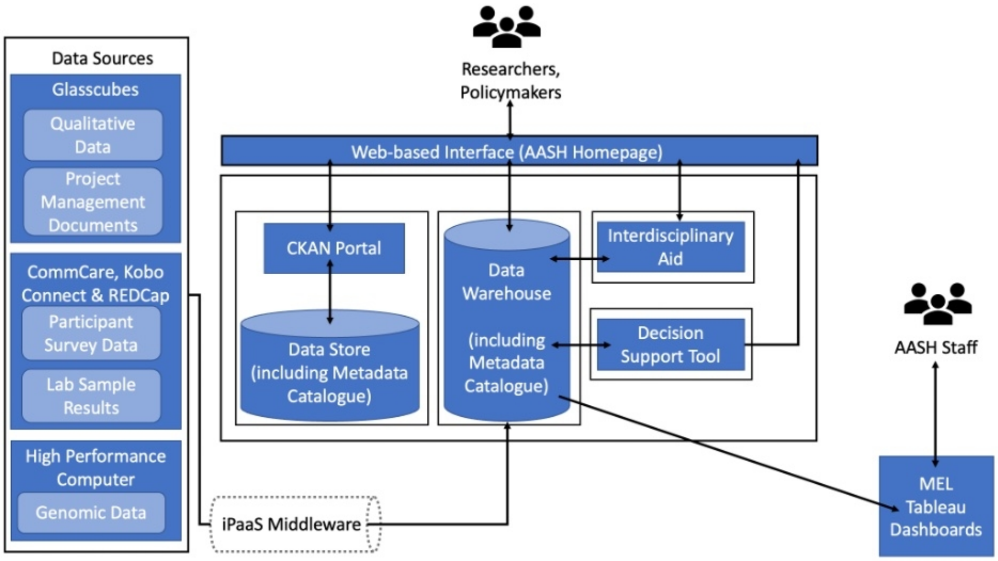

Figure 2: Visualisation of the All-Hub Data Repository (AHDR)

165x92mm (144 x 144 DPI)
